# Moxifloxacin enhances antiproliferative and apoptotic effects of etoposide but inhibits its proinflammatory effects in THP-1 and Jurkat cells

**DOI:** 10.1038/sj.bjc.6603355

**Published:** 2006-10-17

**Authors:** I Fabian, D Reuveni, A Levitov, D Halperin, E Priel, I Shalit

**Affiliations:** 1 Department of Cell and Developmental Biology, Sackler School of Medicine, Tel-Aviv University, Tel Aviv 69978, Israel; 2 Department Microbiology& Immunology, BGU Cancer Research Center, Faculty of Health Sciences, Ben-Gurion University, Beer-Sheva, Israel; 3 Schneider Children's Medical Center of Israel, Petach-Tikva, Israel

**Keywords:** topoisomerase II, chemotherapeutic drugs, angiogenesis, cytotoxicity

## Abstract

Etoposide (VP-16) is a topoisomerase II (topo II) inhibitor chemotherapeutic agent. Studies indicate that VP-16 enhances proinflammatory cytokines secretion from tumour cells, including IL-8, a chemokine associated with proangiogenic effects. Fluoroquinolones inhibit topo II activity in eukaryotic cells by a mechanism different from that of VP-16. The fluoroquinolone moxifloxacin (MXF) has pronounced anti-inflammatory effects *in vitro* and *in vivo*. We studied the effects of MXF and VP-16 on purified human topo II activity and further analysed their combined activity on proliferation, apoptosis and caspase-3 activity in THP-1 and Jurkat cells. Moxifloxacin alone slightly inhibited the activity of human topo II; however, in combination with VP-16 it led to a 73% reduction in enzyme activity. VP-16 inhibited cell proliferation in a time and dose-dependent manner. The addition of moxifloxacin for 72 h to low-dose VP-16 doubled its cytotoxic effect in THP-1 and Jurkat cells (1.8- and 2.6-fold decrease in cell proliferation, respectively) (*P*<0.004). Moxifloxacin given alone did not induce apoptosis but enhanced VP-16-induced apoptosis in THP-1 and Jurkat cells (1.8- and two-fold increase in annexin V positive cells and caspase-3 activity, respectively) (*P*<0.04). VP-16 induced the release of IL-8 in a time and dose-dependent manner from THP-1 cells. Moxifloxacin completely blocked the enhanced release of IL-8 induced by 0.5 and 1 *μ*g ml^−1^ VP-16, and decreased IL-8 release from cells incubated for 72 h with 3 *μ*g ml^−1^ VP-16 (*P*<0.001). VP-16 enhanced the release of IL-1*β* and TNF-*α* from THP-1 cells, whereas the addition of MXF prevented the enhanced cytokine secretion (*P*<0.001). We conclude that MXF significantly enhances VP-16 cytotoxicity in tumour-derived cells while preventing VP-16-induced proinflammatory cytokine release. This unique combination may have clinical benefits and cytotoxic drug ‘sparing effect’ and should be further studied *in vivo*.

Topoisomerase II (topo II) is an important target of chemotherapeutic agents (Berger and Wang, 1996). One of the first drugs to demonstrate an antineoplastic effect through inhibition of topo II was etoposide (VP-16), which prevents resealing of the enzyme-linked DNA breaks (Handle, 1998). Etoposide is used today as frontline therapy for a variety of human malignancies, including leukaemias, lymphomas and several solid tumours (Fry *et al*, 1991; Handle, 1998).

Several studies have indicated that in addition to their known cytotoxic effects, many chemotherapeutic agents, including VP-16, are also prooxidative stressors (Kagan *et al*, 2001) and trigger cytokine production in a variety of cell types *in vitro* (Kawagishi *et al*, 2001; Darst *et al*, 2004). These findings were supported by reports of significant levels of proinflammatory cytokines in patients undergoing chemotherapy for a variety of tumours (Villani *et al*, 2002). The role of proinflammatory cytokines in tumour progression, angiogenesis and metastases has been elucidated in recent years. Specifically, IL-8 was shown to be a potent proangiogenic factor associated with increased microvessel count, increased VEGF expression and poor prognosis in oesophageal squamous cell carcinoma, colonic cancer cells and pancreatic adenocarcinoma cells (Mizukami *et al*, 2005; Ren *et al*, 2005; Trevino *et al*, 2005). Various studies have shown that blocking IL-8 expression reversed many of these proangiogenic and metastatic cell activities, further supporting the important role of this proinflammatory cytokine in tumour progression ((Mizukami *et al*, 2005; Ren *et al*, 2005; Trevino *et al*, 2005).

Combination chemotherapy is commonly employed for treatment of many neoplastic diseases and is considered to provide several advantages over single-agent regimens.

Fluoroquinolones are highly effective antibiotics with a broad antibacterial spectrum (Bakshi *et al*, 2001). At high concentrations, some fluoroquinolones exhibit genotoxic effects in eukaryotic systems as a result of topo II inhibition (Robinson *et al*, 1991). Quinolone-induced inactivation of topo II*α* at high concentrations was proposed to involve the direct binding of quinolones to DNA and their mode of inhibition was shown to be distinct from the inhibitory mechanism of agents like VP-16 (Bromberg *et al*, 2003a). Additional studies suggest that quinolones such as CP-115 953 enhance the formation of double-stranded DNA breaks by human topo II*α* and exert cytotoxic activity in Chinese hamster ovary cells (Elsea *et al*, 1997). Our group and others assessed the *in vitro* activity of certain quinolones against various tumour cell lines. Ciprofloxacin was found to inhibit tumour cell growth of bladder transitional cell carcinoma, colon cancer and prostate cancer cell lines at concentrations achievable with its oral administration (Zehavi-Willner and Shalit, 1992; Shalit *et al*, 1995; Herold *et al*, 2002; Aranha *et al*, 2003).

We have previously shown that the fluoroquinolone moxifloxacin (MXF) inhibits nuclear factor kappa B (NF-κB) activation, mitogen-activated protein kinase activation and synthesis of the proinflammatory cytokines IL-8, TNF-*α* and IL-l*β* in activated human monocytic cells (Weiss *et al*, 2004). It also had a protective anti-inflammatory effect *in vivo* in a model of *Candida albicans* pneumonia in immune suppressed animals, resulting in enhanced survival and reduction in IL-8 and TNF-*α* in lung homogenates (Shalit *et al*, 2002). In the present study, we investigated the effect of MXF alone and in combination with VP-16 on isolated human topo II*α* activity and further studied the effect of the combination on cell proliferation, cytotoxicity and apoptosis in two tumour-derived cell lines, THP-1 and Jurkat. In parallel, we investigated the effect of MXF on VP-16-induced release of proinflammatory cytokines, including IL-8, in these cells.

## MATERIALS AND METHODS

### Human topo II assay

A previously described method was used with slight modifications (Bendetz-Nezer *et al*, 2004). Purified human DNA topo II (p170) (0.5–2 units) (TopoGen Inc., Port Orange, FL, USA) was added to a topo II reaction mixture containing, at a final volume of 20 *μ*l, 50 mM Tris-HCl, (pH 8), 0.5 mM dithiothreitol (DTT), 120 mM KCl, 10 mM MgCl_2_, 0.5 mM EDTA, 25 *μ*g ml^−1^ bovine serum albumin, 1 mM ATP and 250–750 ng of pUC 19 supercoiled DNA plasmid (MBI Fermentas, Hanover, MD, USA). Different concentrations of MXF (20–100 *μ*g ml^−1^) and VP-16 (5–10 *μ*g ml^−1^) were added. After incubation at 37°C for 10 min, the reaction was terminated by adding 5 *μ*l of stopping buffer (final concentration, 1% SDS, 15% glycerol, 0.5% bromphenol blue and 50 mM EDTA, pH 8). The reaction products were analysed by electrophoresis on 1% agarose gel using a Tris-borate/EDTA buffer (89 mM boric acid and 62 mM EDTA) at 1 V cm^−1^, stained with ethidium bromide (1 *μ*g ml^−1^), and photographed using a short-wavelength UV lamp (ChemiImager 5500; Alpha Innotech, San Leandro, CA, USA).

### Cell lines

Human acute monocytic leukaemia cell line THP-1 (ATCC TIB 202) and human acute T-cell leukaemia Jurkat cells were maintained in RPMI 1640 medium supplemented with 10% heat-inactivated fetal bovine serum, 2 mM L-glutamine, 100 units ml^−1^ penicillin and 100 *μ*g ml^−1^ streptomycin at 37°C in a humidified incubator with 5% CO_2_.

### Cytotoxicity assay

THP-1 and Jurkat cells, cultured as described above, were seeded on 96-well plates (at a concentration of 5 × 10^4^ THP-1 cells per 0.1 ml well^−1^ and 2.5 × 10^4^ Jurkat cells per 0.1 ml well^−1^) in triplicate, and various concentrations of MXF, VP-16 and their combination were added. The cells were incubated for 24–72 h at 37°C in 5% CO_2_ atmosphere. For the last 3 h of incubation, MTT (3-(4,5-dimethylthiazol-2-yl)-2,5-diphenyltetrazolium) (5 mg ml^−1^) in phosphate-buffered saline (PBS) was added to each well. The cells were incubated at 37°C for 3 h and 0.04 M HCl was added to dissolve the formazan crystals. The absorbance was then measured at 560 nm with a spectrophotometer (ELISA Reader Molecular Devices Corporation, Sunnyvale, CA, USA).

### Apoptosis assay

Apoptosis was measured by flow cytometry after concurrent staining with fluorescein-conjugated annexin V and propidium iodide (PI), as previously described (Gross-Fischer and Fabian, 2002). In brief, following incubation of THP-1 and Jurkat cells with 1 *μ*g ml^−1^ VP-16 and various concentrations of MXF and their combination for 24 h, cells were collected and washed with cold PBS and resuspended in annexin V-PI binding buffer (10 mM HEPES, pH 7.4, 140 mM NaCl, 2.5 mM CaCl_2_). An aliquot of 100 *μ*l was removed and mixed with 4 *μ*l of annexin V-PI. The mixture was incubated for 15 min at room temperature (RT) in the dark. The cells were then washed once with binding buffer, resuspended in binding buffer and subjected to flow cytometric analysis on FACScan (Becton Dickinson, Franklin Lakes, NJ, USA).

### Fluorogenic assay for caspase-3 activity

Caspse-3 was measured as previously described by us (Gross-Fischer and Fabian, 2002). THP-1 and Jurkat cells were incubated for 24 h with 1 and 3 *μ*g ml^−1^ VP-16 or various concentrations of MXF, or combination of both drugs. Following incubation, the cells were collected, washed, resuspended in 50 mM HEPES (pH 7.4), 0.1% CHAPS, 5 mM DTT and 0.1 mM EDTA, incubated for 15 min on ice and lysed by three successive freeze–thaw cycles at dry ice/37°C. Cell lysates were centrifuged at 14 000 r.p.m. for 15 min, and the supernatants were stored at −70°C. The protein concentration of each sample was estimated using the Bradford Bio-Rad protein assay. For caspase-3 activity, a total of 25 *μ*g protein was incubated with 30 mM ac-DEVD-AMC (BIOMOL Research Laboratories, Plymouth Meeting, PA, USA) at 37°C, for 60 min in the dark. The release of 7-amino-4-methylcoumarine was monitored by a spectrofluorometer using an excitation wavelength of 360 nm and an emission wavelength of 460 nm. In some experiments, a caspase-3 inhibitor (Z-DEVD-FMK, Calbiochem, Nottingham, UK) was added directly to the medium 30 min before the addition of 1 *μ*g ml^−1^ VP-16.

### Western blot analysis of caspase-3

For caspase-3 immunoblotting, total cell lysates were prepared as described before with slight modifications. Cells were collected and washed twice in ice-cold PBS, and then lysed with a solubilising solution on ice for 30 min (Gross-Fischer and Fabian, 2002). The extracts were cleared by centrifugation. Equal amounts of protein (50 *μ*g) were subjected to electrophoresis on 12% SDS–polyacrylamide gel and then electrophoretically transferred to a nitrocellulose membrane. The membranes were incubated with anti-caspase-3 polyclonal antibody (diluted 1:1000, PharMingen, Franklin Lakes, NJ, USA). Actin levels were also assessed as a loading control using an antibody (Santa Cruz Biotechnology, Santa Cruz, CA, USA) that reacts with a broad range of actin isoforms. The blots were then incubated with a secondary antibody, horseradish peroxidase-linked anti-mouse IgG (Santa Cruz Biotechnology). After 1 h at RT and three washes in TBST, the blots were incubated in enhanced chemiluminescence reagent (ECL, Amersham Pharmacia Biotech, Uppsala, Sweden). Bound antibodies were visualised following chemiluminescence detection on autoradiographic film.

### IL-8, IL-1*β* and TNF-*α* production analysis by ELISA

THP-1 cells suspended in RPMI medium, as described above, were placed in 24-well culture plates at a concentration of l × 10^6^ cells ml^−1^ (for the determination of IL-8) and incubated for 24–72 h with various concentrations of VP-16 in the presence or absence of 5–20 *μ*g ml^−1^ MXF. For the determination of IL-1*β* and TNF-*α*, cells were plated at concentrations of l × 10^6^ cells ml^−1^ and 1.5 × 10^6^ cells ml^−1^, respectively, for 24 h in the presence of VP-16 and MXF as described above. Cell-free supernatants were recovered, and the concentrations of IL-8, IL-1*β* and TNF-*α* were determined using ELISA (R&D Systems Inc., Minneapolis MN, USA). The sensitivity of the assay for IL-8 is >10 pg ml^−1^, for IL-1*β* >4 pg ml^−1^ and for TNF-*α* >15 pg ml^−1^. Jurkat cells were pretreated for 1 h with 1–10 ng ml^−1^ phorbol myristate acetate (PMA) and 5–500 ng ml^−1^ ionomycin (Sigma Chemical Co., St Louis, MO, USA) to promote cytokine production (Pestka *et al*, 2005). Various concentrations of VP-16 and MXF were added and the concentrations of IL-8, IL-1*β* and TNF-*α* were determined as described above.

### Statistical analysis

Statistical significance was determined by paired *t*-test (for MTT, fluorogenic assay for caspase-3 activity and for cytokine secretion) and ANOVA: two-factor without replication test (for the annexin-PI studies). A *P*-value of ⩽0.05 was considered significant.

## RESULTS

### Purified human topo II DNA relaxation activity assay

**Figure 1:**
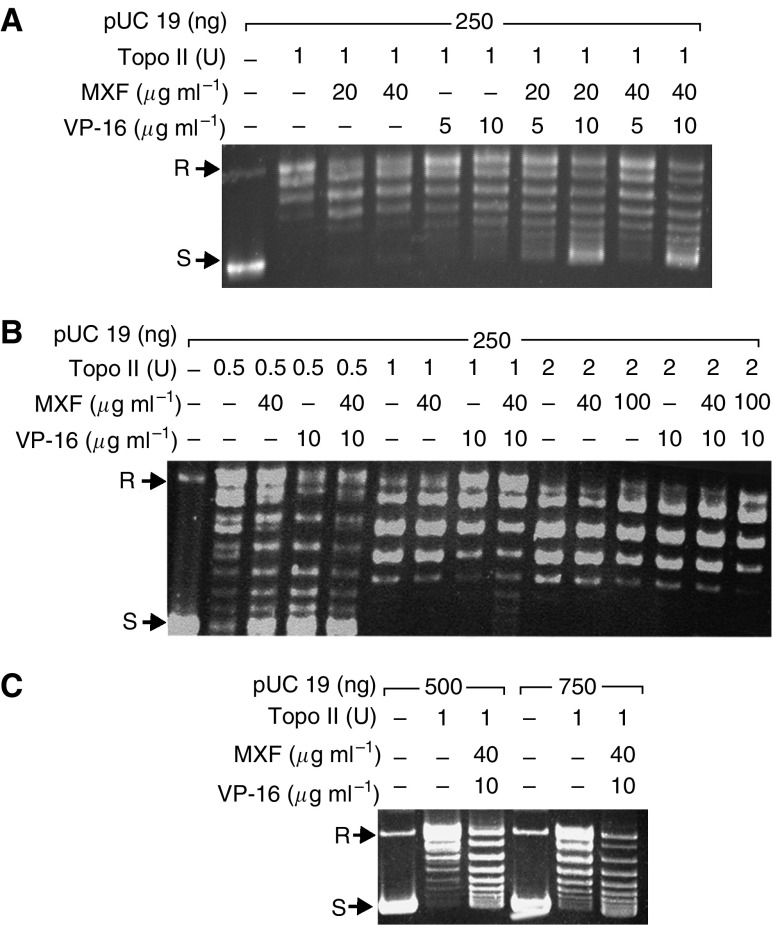
(**A**) Representative agarose gel electrophoresis analysis of the topo II reaction products, obtained with increasing amounts of MXF, VP-16 or their combination. The pUC supercoiled DNA plasmid and the relaxed forms are shown. (**B**) Increasing amounts of topo II (0.5–2 units) were added to reaction mixtures containing a constant amount of pUC19 DNA (250 ng) and 10 *μ*g ml^−1^ VP-16+40 or 100 *μ*g ml^−1^ MXF. (**C**) Increasing amounts of pUC-19 DNA (500–750 ng) were added to topo II reaction mixture containing constant amounts of topo II (1 unit) and 10 *μ*g ml^−1^ VP-16+40 *μ*g ml^−1^ MXF. R=relaxed DNA, S=supercoiled DNA, u=units.

The inhibition of the DNA relaxation activity of human topo II by various concentrations of MXF and VP-16 was investigated using purified human topo II added to a specific reaction mixture containing ATP and supercoiled pUC 19 DNA as the substrate, as described in Materials and Methods. As shown in Figure 1A, MXF at a concentration of 20 and 40 *μ*g ml^−1^ caused a 5–6% inhibition of topo II activity (lanes 3 and 4, respectively). Etoposide reduced topo II activity only slightly at a concentration of 10 *μ*g ml^−1^ (10%) (lane 6), and not at all at a lower concentrations (5 *μ*g ml^−1^) (lane 5). By contrast, the combination of 20 *μ*g ml^−1^ MXF with 5 or 10 *μ*g ml^−1^ VP-16 markedly reduced topo II activity, by 20% (lane 7) and 67% (lane 8), respectively. The combination of a higher concentration of MXF (40 *μ*g ml^−1^) with VP-16 revealed only a slight further inhibition of topo II activity (up to 73%) (lane 10).

The observed increased inhibitory effect of VP-16 by MXF might be due to (1) a direct effect of MXF on the topo II protein, rendering it more susceptible to the action of VP-16; (2) MXF affects the DNA (e.g. intercalation) in a way that increases the VP-16-induced stabilisation of the DNA–enzyme cleavable complexes. To determine which of the possibilities do occur, we performed two classical biochemical competition-based assays (Aflalo *et al*, 1994). Topoisomerase II activity was measured in the presence of constant amounts of DNA and MXF/VP-16 and increasing amounts of topo II enzyme (Figure 1B) or vice versa; topo II activity was measured in the presence of constant amounts of enzyme and MXF/VP-16 and increasing amounts of DNA (Figure 1C). The results show that only by increasing the amount of topo II enzyme, it is possible to overcome the inhibitory effect of MXF/VP-16 (Figure 1B compared to 1C), suggesting a possible, yet unclear, interaction of MXF with the topo II protein.

On the basis of our observations that MXF enhances the inhibition of purified topo II activity conferred by VP-16, we investigated the possible association between the effect on isolated topo II activity and the cytotoxic activity of the drug combination.

### Effect of MXF on the antiproliferative action of VP-16

**Figure 2:**
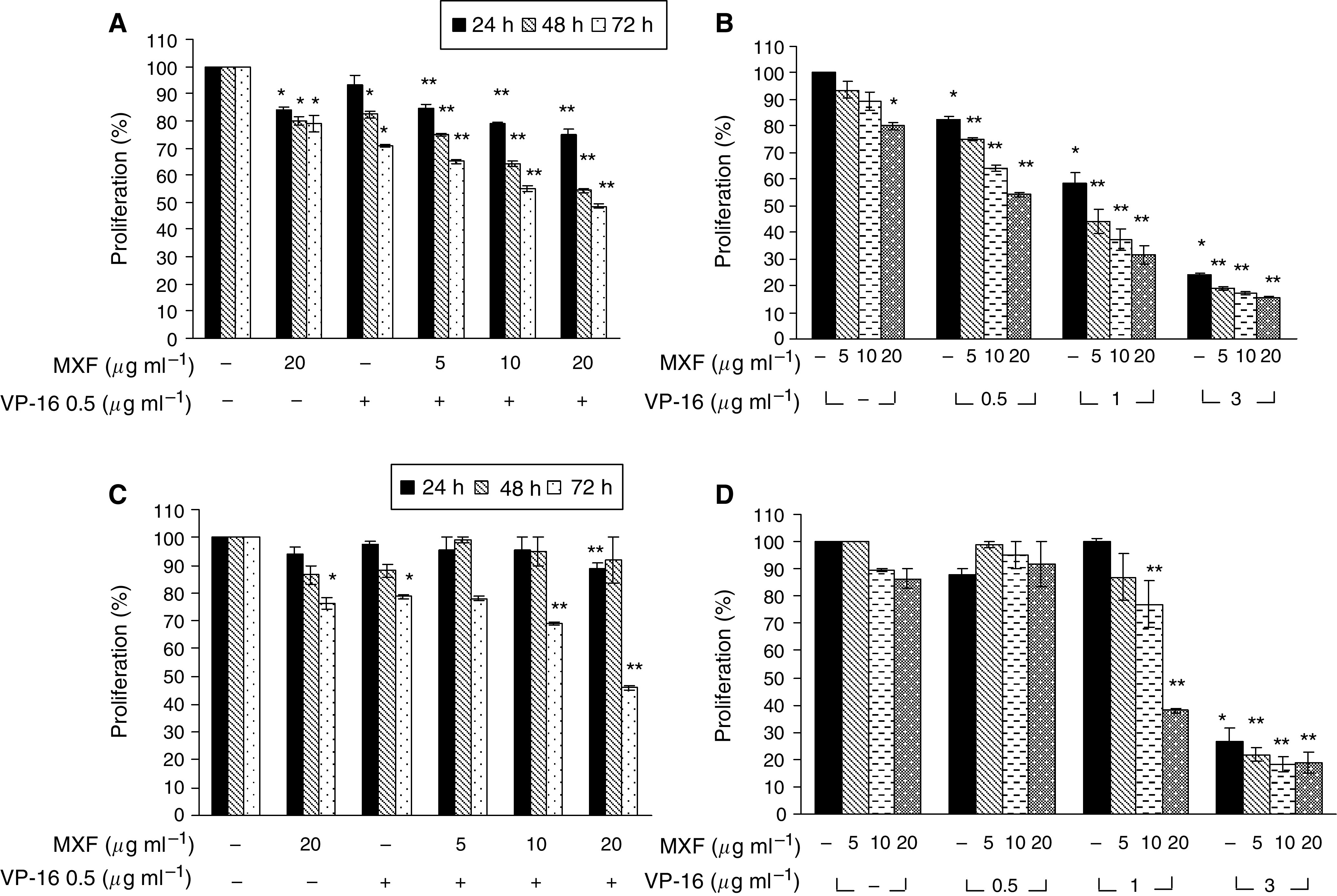
Moxifloxacin enhances the antiproliferative effect of VP-16. Time-dependent studies: THP-1 cells (**A**) and Jurkat cells (**C**) were incubated for 24–72 h with 0.5 *μ*g ml^−1^ VP-16 in the presence or absence of various concentrations of MXF. Cell proliferation was determined by colorimetric MTT assay. Dose-dependent studies: THP-1 cells (**B**) and Jurkat cells (**D**) were incubated for 48 h in the presence of the indicated concentrations of VP-16 and MXF. Cell proliferation was determined as described above. Results are expressed as mean±s.e. of four experiments performed in triplicate. ^*^P<0.05 cells treated with drugs *vs* control. ^**^P<0.007 for cells treated by VP-16+MXF *vs* VP-16 alone.

We performed time-dependent studies on the effect of VP-16 (0.5 *μ*g ml^−1^) on the proliferation of THP-1 cells. Figure 2A indicates that a decrease of 17.7±0.2 and 29±0.3% was observed upon incubation of the cells with the drug for 48 and 72 h, respectively (*P*<0.001). Moxifloxacin given alone, at concentrations of 5 or 10 *μ*g ml^−1^ did not affect cell proliferation (data not shown) whereas incubation of the cells with 20 *μ*g ml^−1^ MXF for 24–72 h resulted in a 16±0.1–20±0.4% inhibition in cell proliferation (*P*<0.05).

A significant decrease in cell proliferation was seen with the combination of 0.5 *μ*g ml^−1^ VP-16 and MXF compared to VP-16 alone. Upon exposure of the cells to a combination of VP-16 and 10 *μ*g ml^−1^ MXF for 48 and 72 h, a marked decrease in cell proliferation (up to 36±0.6 and 45±0.8%, respectively) was observed (*P*=<0.001). Maximum inhibition in cell proliferation was observed upon incubation of the cells with 0.5 *μ*g ml^−1^ VP-16 and 20 *μ*g ml^−1^ MXF for 72 h (up to 52±0.5%), compared to VP-16 alone (*P*<0.001) (Figure 2A).

Dose-dependent studies of VP-16 and MXF were performed, following incubation of the cells for 48 h with the drugs. Exposure of THP-1 cells to VP-16 for 48 h resulted in a concentration-dependent decrease in cell proliferation (Figure 2B). It should be noted that exposure of THP-1 cells to a low dose of the cytotoxic drug VP-16 (0.5 *μ*g ml^−1^) and 20 *μ*g ml^−1^ MXF resulted in a 46% inhibition of cell proliferation, similar to the inhibitory effect of 1 *μ*g ml^−1^ VP-16 alone (41%). Maximal inhibition of cell proliferation was observed upon incubation of the cells with 3 *μ*g ml^−1^ VP-16 and 20 *μ*g ml^−1^ MXF compared to VP-16 alone (85.4±1.6 *vs* 76±1.5%, respectively, *P*=0.006).

In Jurkat cells (Figure 2C), incubation of the cells for 72 h with a low dose of VP-16 (0.5 *μ*g ml^−1^), resulted in a 21±0.6% inhibition of cell proliferation (*P*<0.001). As with THP-1 cells, incubation of Jurkat cells with 5 or 10 *μ*g ml^−1^ MXF for 24–72 h, did not affect cell proliferation (data not shown), whereas incubation for 72 h with 20 *μ*g ml^−1^ MXF, resulted in a 24±0.6% inhibition of cell proliferation (*P*=0.008). Exposure of the cells for 72 h to a combination of VP-16 (0.5 *μ*g ml^−1^) and 10 or 20 *μ*g ml^−1^ MXF, led to 31±0.1 and 54±0.5% inhibition, respectively, compared to 21±0.6% with VP-16 alone (*P*<0.004).

The addition of various concentrations of VP-16 alone reduced cell proliferation in a dose-dependent manner (Figure 2D). Like in THP-1 cells, the addition of increasing concentrations of MXF to Jurkat cells incubated for 48 h with various concentrations of VP-16 further decreased cell proliferation. Incubation of the cells with 1 *μ*g ml^−1^ VP-16 and 20 *μ*g ml^−1^ MXF resulted in 56±5% decrease, compared to 22±2% for VP-16 alone (*P*=<0.001). The maximal inhibition of cell proliferation was observed upon incubation of the cells with 3 *μ*g ml^−1^ VP-16 and 20 *μ*g ml^−1^ MXF (up to 89±8%) (Figure 2D).

### Effect of MXF on VP-16-induced apoptosis

**Figure 3:**
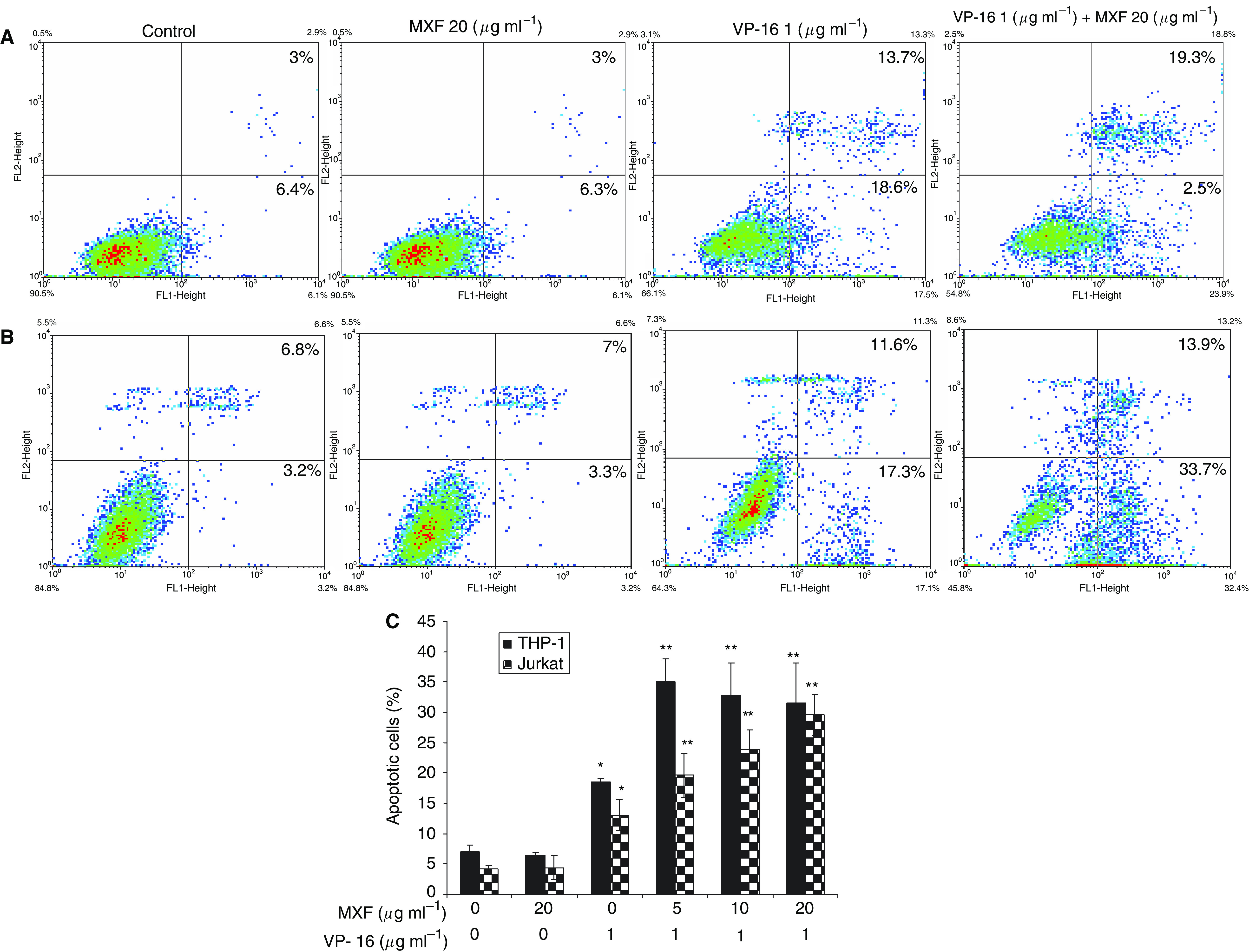
Moxifloxacin enhances apoptosis induced by VP-16. THP-1 (**A** and **C**) and Jurkat cells (**B** and **C**) were incubated for 24 h with VP-16 and MXF as indicated and flow cytometric analysis was performed by binding of annexin V and uptake of PI. A representative experiment is shown in (**A** and **B**). Results (mean±s.e.) of two independent experiments are shown in (**C**). The percentage of annexin V-positive, PI-negative cells is indicated in the lower right quadrangle and of annexin V-positive, PI-positive cells in the upper right quadrangle. The *X*-axis shows log annexin V fluorescence intensity and the *Y*-axis shows PI fluorescence intensity. ^*^P<0.04 for cells treated with VP-16+MXF *vs* VP-16 alone.

Phosphatidylserine, which is normally confined to the inner leaflet of the plasma membrane, is exported to the outer plasma membrane leaflet during apoptosis. We assessed phosphatidylserine externalisation following the exposure of THP-1 and Jurkat cells for 24 h to MXF alone or to 1 *μ*g ml^−1^ VP-16 in the presence or absence of 5–20 *μ*g ml^−1^ MXF. As shown in Figure 3A and C, in the THP-1 cells, MXF (20 *μ*g ml^−1^) given alone, did not affect apoptosis whereas a marked increase in apoptotic (annexin V-positive) cells was observed after exposure to VP-16, and a further 1.7-fold to 1.8-fold increase with the addition of 5–20 *μ*g ml^−1^ MXF (*P*=0.038). As with THP-1 cells, exposure of Jurkat cells to 20 *μ*g ml^−1^ MXF did not affect apoptosis whereas exposure to 1 *μ*g ml^−1^ VP-16 alone (Figure 3B) resulted in a mild increase in apoptotic cells, with a further increase after the addition of MXF, by 1.7-fold for 10 *μ*g ml^−1^ MXF (*P*=0.024), and by 2.2-fold for 20 *μ*g ml^−1^ MXF (*P*=0.015) (Figure 3C).

### Caspase-3 activation

**Figure 4:**
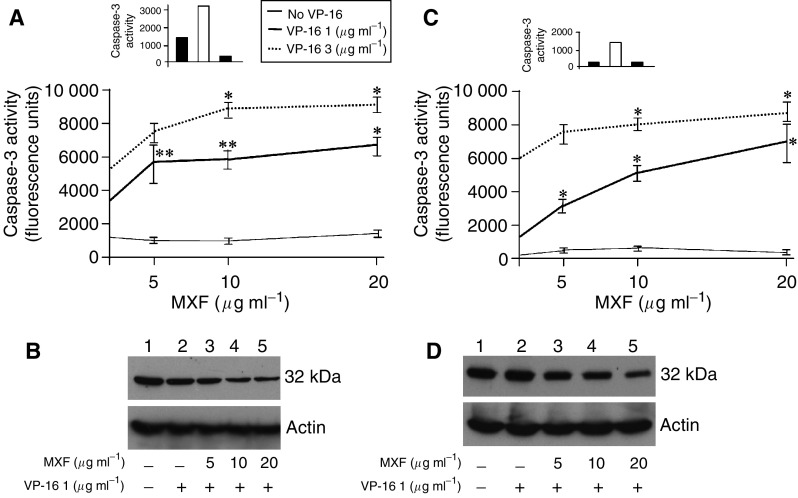
Moxifloxacin enhances caspase-3 activation induced by VP-16. THP-1 (**A**) and Jurkat cells (**C**) were incubated with the indicated concentrations of VP-16 and MXF for 24 h and lysates were prepared. Caspase-3 activity was measured using DEVD-AMC as the substrate. The data represent the mean±s.e. of three experiments. Effect of caspase-3 inhibitor (insert in **A** and **C**): grey column indicates control cells; white column indicates cells treated with 1 *μ*g ml^−1^ VP-16. The black column cells were preincubated for 30 min with Z-DEVD-FMK (caspase-3 inhibitor) before exposure to 1 *μ*g ml^−1^ VP-16. ^*^P<0.05; ^**^P<0.012 VP-16+MXF *vs* VP-16-treated cells. Western blot analysis of procaspase-3. THP-1 (**B**) and Jurkat cells (**D**) were incubated in medium (lane 1) or with 1 *μ*g ml^−1^ VP-16 (lanes 2–5) and MXF (5–20 *μ*g ml^−1^) (lanes 3–5, respectively). Lysates were prepared and samples containing 50 *μ*g protein were resolved in 12% SDS gels and electroblotted onto nitrocellulose membranes. The membranes were probed with anticaspase-3 rabbit polyclonal antibody and with antiactin antibody.

DEVD-AMC is a specific substrate for caspase-3 that mimics the PARP cleavage site. To test caspase-3 activation, cell lysates from THP-1 and Jurkat cells that were treated with VP-16 in the presence or absence of 5–20 *μ*g ml^−1^ MXF for 24 h were incubated with the substrate, and the increase in fluorescence due to enzymatic cleavage of the peptides was measured with a fluorometer. As shown in Figure 4A, in THP-1 cells, treatment with 1 or 3 *μ*g ml^−1^ VP-16 significantly increased caspase-3 activation (*P*=0.011 and *P*=0.044, respectively). The addition of 20 *μ*g ml^−1^ MXF further enhanced this increase by two-fold in the presence of 1 *μ*g ml^−1^ VP-16 (*P*=0.045) and by 1.7-fold in the presence of 3 *μ*g ml^−1^ VP-16 (*P*=0.05). To confirm that the caspase activity observed was specific to caspase-3, we used a caspase-3-specific inhibitor, Z-DEVD-FMK. Treatment of cells with the inhibitor before the addition of 1 *μ*g ml^−1^ VP-16 reduced the degree of caspase-3 activation (insert in Figure 4A).

Immunoblots performed on lysates of these cells demonstrated that MXF enhanced the cleavage of procaspase 3 induced by 1ìg ml^−1^ VP-16 in a dose-dependent manner (Figure 4B).

In Jurkat cells, as shown in Figure 4C, incubation with 1 or 3 *μ*g ml^−1^ VP-16 significantly enhances caspase-3 activity by 4.4-fold (*P*<0.001) and by 21.3-fold (*P*=0.014), respectively. The addition of MXF to VP-16 further enhanced the activity of caspase-3. The addition of 5, 10 or 20 *μ*g ml^−1^ MXF to l *μ*g ml^−1^ VP-16 resulted in a 2.3-, 3.5- and 4.4-fold increase in caspase-3 activity, respectively (*P*=0.028, *P*=0.05 and *P*=0.05, respectively), indicating that MXF increases the inhibition of Jurkat cell growth, induced by VP-16, by induction of apoptosis via activation of caspase-3 activity. Similarly, the combination of 20 *μ*g ml^−1^ MXF and 3 *μ*g ml^−1^ VP-16 led to an additional 1.45-fold increase in caspase-3 activity (*P*=0.035) (Figure 4C). Treatment of the cells with Z-DEVD-FMK before the addition of 1 *μ*g ml^−1^ VP-16 reduced the degree of caspase-3 activation (inset in Figure 4C), confirming that the caspase activity observed was specific to caspase-3. Immunoblots performed on lysates of these cells demonstrated that MXF enhanced the cleavage of procaspase-3 induced by 1 *μ*g ml^−1^ VP-16 in a dose-dependent manner (Figure 4D).

### Secretion of proinflammatory cytokines

**Figure 5:**
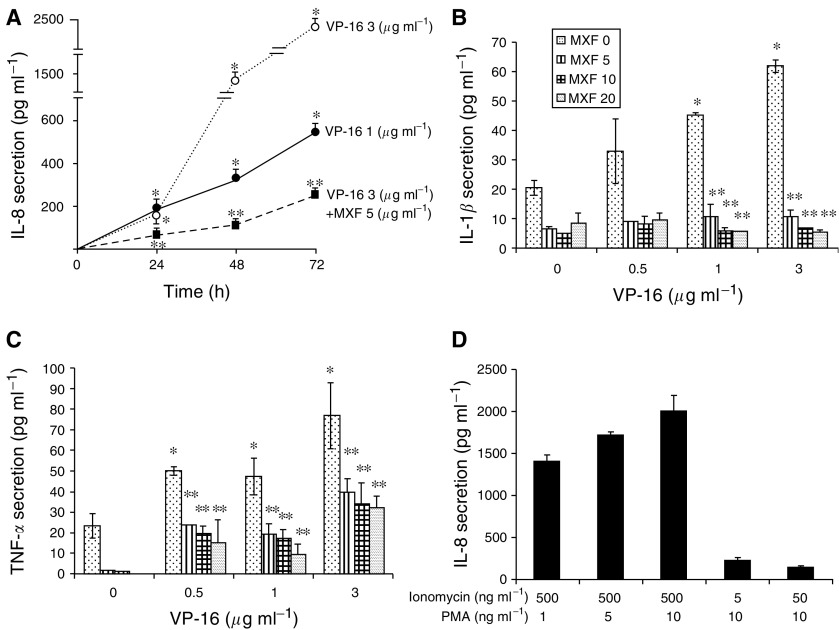
Moxifloxacin decreases proinflammatory cytokine secretion induced by VP-16 in THP-1 cells. Time-dependent studies: THP-1 cells were incubated for 24–72 h with increasing concentrations of VP-16 and 5 *μ*g ml^−1^ MXF as indicated. The concentrations of IL-8 (**A**) were measured by ELISA. Dose-dependent studies: THP-1 cells were incubated for 24 h with increasing concentrations of VP-16 and MXF. The concentrations of IL-1*β* (**B**) and TNF-*α* (**C**) in culture supernatant were measured by ELISA. The values are the mean±s.e. of four experiments performed in duplicates. ^*^P<0.018; ^**^P<0.008 VP-16+MXF *vs* VP-16-treated cells. (**D**) Jurkat cells were incubated for 24 h with the indicated concentrations of PMA and ionomycin and the concentration of IL-8 was measured by ELISA. The values are the mean±s.e. of four experiments performed in duplicate.

We investigated the effect of VP-16 on the secretion of proinflammatory cytokines by THP-1 and Jurkat cells. The spontaneous release of IL-8 by THP-1 cells cultured for 72 h was 61±4 pg ml^−1^. The addition of 5 *μ*g ml^−1^ MXF resulted in a three-fold decrease in the spontaneous release of the cytokine (to 19.3±1.2 pg ml^−1^) (*P*<0.001). Time-dependent studies performed with THP-1 cells indicate that exposure of the cells to 1 *μ*g ml^−1^ VP-16 for 24–72 h resulted in a 3.1–8.9-fold increase in IL-8 secretion (*P*<0.001) (Figure 5A). The addition 5 *μ*g ml^−1^ MXF to cells cultured for 72 h with 1 *μ*g ml^−1^ VP-16 completely abolished the increase in IL-8 secretion induced by VP-16 (*P*<0.001) (data not shown). Exposure of the cells for 24–72 h to 3 *μ*g ml^−1^ VP-16 resulted in additional increase in IL-8 secretion up to 2318±70 pg ml^−1^ (at 72 h) (*P*<0.001) (Figure 5A). The addition of 5 *μ*g ml^−1^ MXF to cells cultured with 3 *μ*g ml^−1^ VP-16 induced a time-dependent decrease in IL-8 secretion induced by VP-16 (up to 10-fold decrease at 72 h) (*P*<0.001). No additional decrease in IL-8 secretion was observed in the presence of 10 or 20 *μ*g ml^−1^ MXF (Figure 5A). Dose-dependent studies of VP-16 on the secretion of IL-l*β* and TNF-*α* were performed. Exposure of THP-1 cells for 24 h to 1 or 3 *μ*g ml^−1^ VP-16 induced an increase in the secretion of IL-l*β* and TNF-*α* (Figure 5B and C, respectively). The addition of MXF, even at low concentration (5 *μ*g ml^−1^), completely inhibited the enhanced IL-l*β* and TNF-*α* secretion induced by 0.5 and 1 *μ*g ml^−1^ VP-16. The addition of 5, 10 or 20 *μ*g ml^−1^ MXF to cells incubated with 3 *μ*g ml^−1^ VP-16 decreased TNF-*α* secretion by 52%, 44% and 66%, respectively (*P*=<0.001 for each concentration) (Figure 5C). In Jurkat cells, no secretion of IL-8 could be detected without stimulation with PMA and ionomycin (data not shown). Pretreatment of the cells with 500 ng ml^−1^ ionomycin and 1–10 ng ml^−1^ PMA for 24 h resulted in a dose-dependent increase in IL-8 secretion (Figure 5D). Lower concentrations of IL-8 were detected upon pretreatment with 10 ng ml^−1^ PMA and 5 or 50 ng ml^−1^ ionomycin (Figure 5D). The addition of 1–3 *μ*g ml^−1^ VP-16 to Jurkat cells pretreated with PMA at a dose of 10 ng ml^−1^ or lower and 500 ng ml^−1^ ionomycin or lower had no effect on IL-8 release from the cells (data not shown).

## DISCUSSION

Several studies on the antiproliferative activity of fluoroquinolone antibiotics have shown that quinolones such as ciprofloxacin and fleroxacin inhibit the growth of various human tumour cells, including transitional cell carcinoma of the bladder and human colorectal carcinoma cells (Miclau *et al*, 1998; Aranha *et al*, 2000). El-Rayes *et al* (2002) reported that ciprofloxacin acts synergistically with VP-16 in hormone-resistant prostate cancer cells and Kamat *et al* (1999) demonstrated that ciprofloxacin and ofloxacin exert synergistic activity with doxorubicin in bladder cancer cell lines.

The present study investigated, for the first time, the effect of MXF in combination with VP-16 on the activity of human topo II by measuring the relaxation of supercoiled pUC 19 DNA plasmid. We have also defined the functional interaction of the drugs by investigating their effect on the cytotoxic activity towards THP-1 and Jurkat cells.

We found that MXF alone (at a concentration of 20 or 40 *μ*g ml^−1^) only slightly inhibited human topo II activity, but in combination with VP-16 it led to a significant increase in the inhibitory effect of the anticancer drug on topo II activity. In addition, we found that the inhibitory effect of the combined drugs on topo II could be inhibited only by increasing the amount of the enzyme protein in the reaction and not by adding higher concentrations of the DNA substrate. This suggests that MXF enhances the inhibitory effects of VP-16 on topo II by affecting the enzyme protein in such a way that it renders it to become more susceptible to VP-16.

Other investigators (Perrone *et al*, 2002) studied the inhibition of topo II by four quinolones and ultraviolet A irradiation, and found that MXF at various concentration up to 10 *μ*M (equivalent to 4 *μ*g ml^−1^) did not show enzyme inhibitory activity in the absence or presence of UVA irradiation. We believe that the discrepancy between the studies may be explained by the use of different concentrations of the drug.

To the best of our knowledge, the experiments reported here are also the first to examine the interaction of MXF and VP-16 on the cytotoxic activity of VP-16 and the effect of the combination on cytokine release induced by VP-16. Our study revealed that MXF by itself (at 20 *μ*g ml^−1^) induced a slight antiproliferative effect (up to 20% decrease in cell proliferation) on THP-1 or Jurkat cells. An additive effect was observed upon incubation of the cells with VP-16 to significantly decrease cell proliferation. A possible explanation for this additive effect is the different mechanisms of action against topo II conferred by the two drugs. Bromberg *et al* (2003b) have shown that VP-16 acts by inhibiting the ability of topo II to ligate cleaved DNA molecules, whereas quinolones have little effect on ligation but stimulate the forward rate of topo II-mediated DNA cleavage. These two distinct mechanisms may work in concert and lead to the observed additivity in the antiproliferative effects of VP-16 and MXF. Using flow cytometric analysis to determine the mechanism of action of the drugs, we observed that VP-16 induced apoptosis in the two cell lines and that MXF potentiated this apoptotic effect. This finding was supported by measuring levels of caspase-3, which is activated during the process of apoptosis and is one of the key enzymes required for the execution of the apoptotic programme. The results showed that MXF significantly enhanced VP-16-induced activation of caspase-3 in THP-1 and Jurkat cells and that its effect was dose dependent. Western blot analysis confirmed the enhanced proteolytic cleavage of procaspase-3 induced by the combination of MXF and VP-16. Together, these observations indicate that MXF acts as a potentiating drug with VP-16 to enhance VP-16's cytotoxic effect and tumour lysis via activation of caspase-3 activity.

An important observation is the fact that 0.5 *μ*g mg^−1^ VP-16 combined with 20 *μ*g mg^−1^ of MXF led to the same inhibition of cell proliferation as a double dose of VP-16 (1 *μ*g ml^−1^) alone. This may imply a cytotoxic-drug ‘sparing effect’ by MXF. The translation of this phenomenon in the clinical setting is that instead of increasing the dose of the cytotoxic agent, along with its associated toxic side effect, one may use a lower dose of the cytotoxic agent and add the antimicrobial agent MXF with its excellent safety profile, and obtain the same antitumour effects with much less toxicity and adverse effects. It should also be noted, that the concentration of MXF cited above is readily attainable in various tissues such as colon, bladder, prostate and lung cells following the commonly used 400 mg daily oral dose of MXF.

We show in the present study that treatment of THP-1 cells with VP-16 induced the release of the proinflammatory cytokines IL-8, IL-1*β* and TNF-*α*. Recent studies have shown that IL-8 is a proangiogenic cytokine regulating tumorigenesis in DLD-1 colon cancer cells (Mizukami *et al*, 2005), and that it also serves as an autocrine growth factor in human colon carcinoma cells *in vitro* (Brew *et al*, 2000). These effects should be looked at as undesired side effects of the drug. Chemotherapy and radiotherapy are prescribed to cancer patients in the hope that dying cells will be safely scavenged by phagocytic cells, such as macrophages. However, *in vitro* and *in vivo* studies showed that phagocytosis of VP-16-treated P388 cells by macrophages was associated with the release of IL-8 and other cytokines, such as MIF and MIP-2 (Kawagishi *et al*, 2001). In addition, VP-16 and the chemotherapeutic agent mitomycin were also found to induce IL-8 and TNF-*α* production by a human epithelial carcinoma cell line (KB cells) that expressed platelet-activating factor receptor (Darst *et al*, 2004). The enhanced expression of cytokines induced by VP-16 may in part be associated with certain side effects and should be looked upon with caution owing to the associated proangiogenic activity of IL-8. Our results showed that MXF significantly inhibited the VP-16-enhanced production of IL-8, TNF-*α* and IL-1*β* in THP-1 cells, but not in Jurkat cells. Accordingly, Aceves *et al* (2004) reported a different pattern of gene expression in THP-1 and Jurkat cells on their exposure to chemotherapeutic drugs. This may suggest that the inhibitory effect of MXF on the release of proinflammatory cytokines by cells is tumour cell specific.

The inhibitory effect of MXF on cytokine secretion from THP-1 cells also confirms our previous observations of MXF inhibition of the synthesis of proinflammatory cytokines in THP-1 cells and human peripheral blood monocytes stimulated with LPS-phorbol myristate acetate (Weiss *et al*, 2004) or *Aspergillus fumigatus* (Shalit *et al*, 2006). Additionally, MXF inhibited NF-κB and mitogen-activated protein kinase activation in THP-1 cells and in a human respiratory epithelial cell line (Weiss *et al*, 2004; Werber *et al*, 2005). Using a murine model of *Candida* pneumonia in immune-suppressed animals, we found that MXF exerted a protective anti-inflammatory effect, resulting in a marked decrease in bronchopneumonia and enhanced survival. This protective efficacy was associated with a significant reduction in IL-8 and TNF-*α* in lung homogenates and an inhibition of NF-κB nuclear mobilisation in alveolar macrophages and lung epithelial cells (Shalit *et al*, 2002).

Nuclear factor-κB is a well-known mediator of inflammatory cytokines (Won *et al*, 2005), and it was shown to be activated during apoptosis following cell incubation with a variety of agents including VP-16 (Usami *et al*, 1998). Based on our previous and current observations, it seems plausible that MXF decreases VP-16-induced proinflammatory cytokine secretion in THP-1 cells via inhibition of NF-κB activation.

In summary, this study demonstrates an important role for MXF in enhancing the cytotoxic effects of VP-16 whereas, at the same time, decreasing VP-16-induced proinflammatory cytokine secretion from cells, which may be harmful during chemotherapeutic treatment. Our results suggest that MXF may be a valuable new addition to chemotherapeutic armamentarium, simultaneously improving the cytotoxic activity while reducing the side effects of VP-16 and similar agents.
